# Case Report: Secondary neurolymphomatosis successfully treated with sequential Bruton’s tyrosine kinase inhibitor and bispecific antibody therapy

**DOI:** 10.3389/fonc.2026.1738551

**Published:** 2026-03-12

**Authors:** Mitsuaki Oura, Masanori Toho, Daisuke Ikeda, Naoya Fukuda, Ana Salinas Torres, Fuminari Fujii, Hajime Sakuma, Atsushi Uehara, Rikako Tabata, Kentaro Narita, Masami Takeuchi, Kosei Matsue

**Affiliations:** Division of Hematology/Oncology, Kameda Medical Center, Kamogawa, Chiba, Japan

**Keywords:** bispecific antibody, Bruton’s tyrosine kinase inhibitor, case report, chemotherapy, diffuse large B-cell lymphoma, epcoritamab, ibrutinib, neurolymphomatosis

## Abstract

**Background:**

Neurolymphomatosis frequently impairs physical function, rendering patients unable to tolerate chimeric antigen receptor T-cell therapy (CAR-T). An alternative treatment strategy which can cross the blood-nerve barrier is warranted.

**Case:**

A 64-year-old woman had a history of *MYD88L265P* mutated diffuse large B-cell lymphoma (DLBCL) successfully treated with Pola-R-CHP plus high-dose methotrexate one year prior. However, she developed progressive muscle weakness in her limbs, with a three-month history. Upon admission, she was bedridden, unable to resist gravity, and experienced bladder and rectal disturbances. Imaging studies revealed neurolymphomatosis involving the bilateral trigeminal nerves, cervical/brachial plexus, brachial nerves, lumbosacral plexus, and femoral nerves. Ibrutinib 560 mg/day combined with rituximab led to complete remission, and she regained the ability to walk within three months. Unfortunately, neurolymphomatosis relapsed after six months of ibrutinib treatment. Epcoritamab led to another complete remission, with a progression-free survival of six months. The adverse events were manageable, including Grade 1 cytokine release syndrome.

**Conclusion:**

This report is the first to demonstrate the effectiveness of epcoritamab in treating neurolymphomatosis. Bispecific antibodies may serve as a valuable bridging therapy for CAR-T, helping to restore their physical function. Bruton’s tyrosine kinase inhibitors could also be an option for *MYD88L265P* mutated disease.

## Introduction

Neurolymphomatosis is a rare manifestation of non-Hodgkin lymphoma, in which lymphoma cells infiltrate the peripheral nerves (1, 2). Due to its rarity, neurolymphomatosis has been a poor prognosis disease with no standard therapy. Most patients with neurolymphomatosis have received high-dose methotrexate (HD-MTX)--based systemic chemotherapy regimens without sustained efficacy (3–5). In these studies, the reported progression-free survival (PFS) with HD-MTX regimens ranged from six to fourteen months. In a recent systematic review compiling 459 cases of neurolymphomatosis from 264 studies, more than half of the patients received MTX-based therapy, with a median overall survival (OS) of 18 months; notably, the prognosis was poorer in secondary neurolymphomatosis, with a median OS of only 13 months (6). Recently, chimeric antigen receptor T-cell (CAR-T) therapy has emerged as a novel and potentially effective option for secondary neurolymphomatosis, with seven of eleven reported patients responding and three achieving complete remission (CR) (7). Given the potential for substantial CAR-T-associated toxicity, patients with declined performance status are often ineligible for this therapy (7, 8), highlighting the need to further explore novel treatment options for neurolymphomatosis. We report a case of secondary neurolymphomatosis characterized by extremely low physical function, in which sequential therapy with the Bruton’s tyrosine kinase inhibitor (BTKi) ibrutinib and the CD20-CD3 bispecific antibody epcoritamab led to significant clinical recovery with manageable adverse effects. This is the first report of epcoritamab showing efficacy against neurolymphomatosis.

## Case description

**Figure 1:**
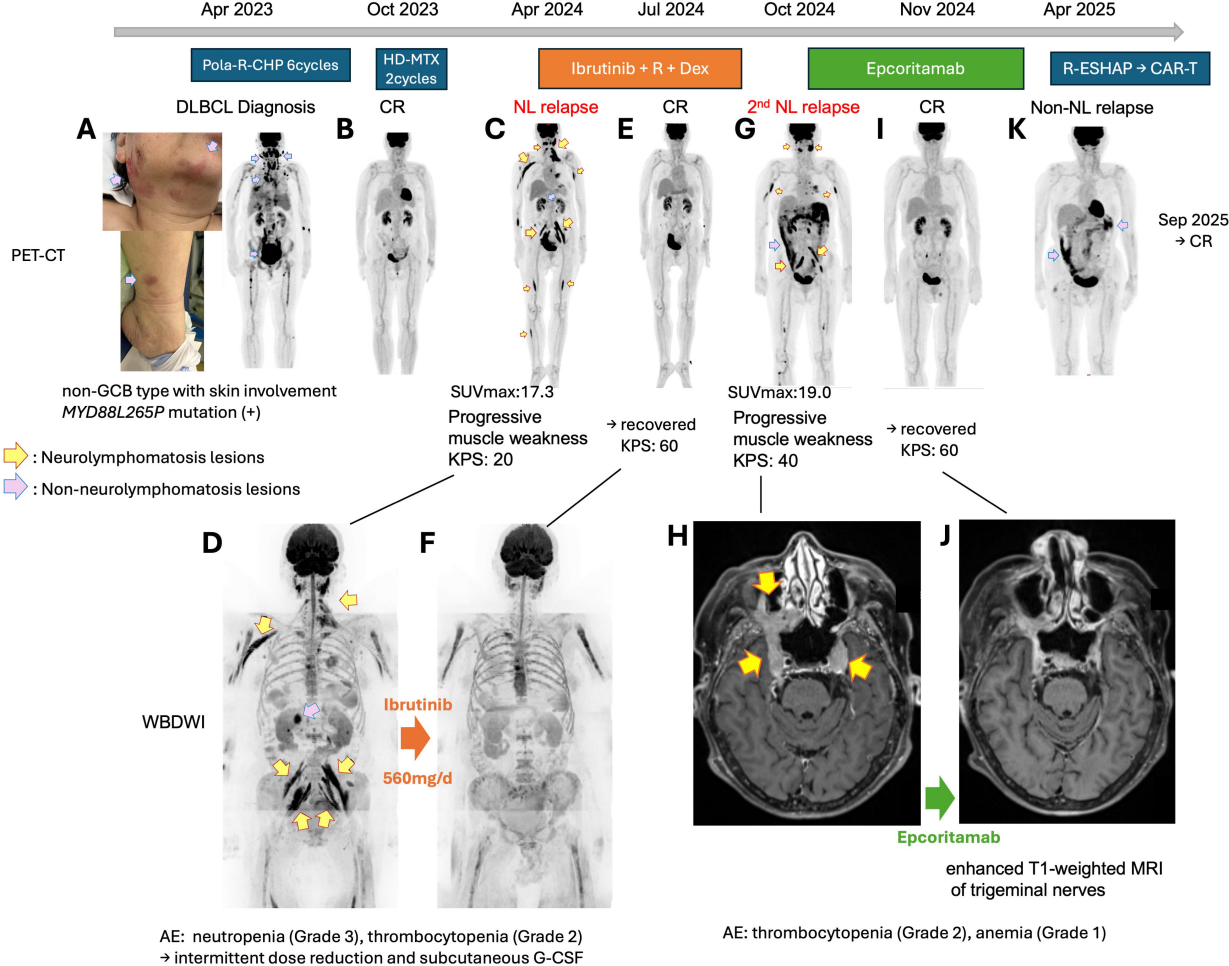
PET-CT images and clinical course. **(A)** Skin rash and PET-CT imaging of DLBCL when it was first diagnosed. There were lesions in the lymph nodes and skin. At this time, the patient did not yet have neurolymphomatosis. Non-neurolymphomatosis lesions are indicated with pink arrows. **(B)** PET-CT images after six courses of R-Pola-CHP when the patient achieved CR. **(C)** PET-CT image at the first relapse. Yellow arrows indicate neurolymphomatosis lesions. **(D)** WBDWI image at the first relapse. **(E)** PET-CT image of the patient achieving CR after ibrutinib + R + Dex treatment. **(F)** WBDWI image of the patient achieving CR after ibrutinib + R + Dex treatment. **(G)** PET-CT image of the second relapse as neurolymphomatosis. Intra-abdominal soft tissue accumulation of ^18^F-fluorodeoxyglucose was also prominent. **(H)** Enhanced T1-weighted MRI image of trigeminal nerves in the second relapse as neurolymphomatosis. **(I)** PET-CT image of the patient achieving CR during epcoritamab treatment. **(J)** Enhanced T1-weighted MRI image of trigeminal nerves in CR during epcoritamab treatment. **(K)** PET-CT image of the non-neurolymphomatosis relapse. Pola-R-CHP, polatuzumab vedotin, rituximab, cyclophosphamide, doxorubicin and prednisolone; HD-MTX, high-dose methotrexate; R-ESHAP, rituximab, etoposide, dexamethasone, cytarabine, cisplatin; NL, neurolymphomatosis; CR, complete remission; R+Dex, rituximab and dexamethasone; PET-CT, positron emission tomography-computed tomography; GCB, germinal center B-cell; SUV, standardized uptake value; KPS, Karnofsky Performance Status; WBDWI, whole-body diffusion weighted MRI; MRI, magnetic resonance imaging; G-CSF, granulocyte colony stimulating factor.

A 64-year-old woman developed muscle weakness and tingling pains in her limbs, which progressed over three months. She had a history of *MYD88L265P*-mutated diffuse large B-cell lymphoma (DLBCL) one year prior with extensive skin involvement, which might have represented extranodal tropism (**Figure 1A**). Immunohistochemistry of a cutaneous lesion showed the non-GCB phenotype (CD20+, CD5-, CD10-, BCL2+, BCL6+, MUM1+, C-MYC+, Mib-1 index high (80%), **Supplementary Figure 1**). BCL2 split and C-MYC split were negative after fluorescent *in situ* hybridization. This condition had been successfully treated with six courses of Pola-R-CHP (polatuzumab vedotin, rituximab, cyclophosphamide, doxorubicin, and prednisone) and two courses of HD-MTX (**Figures 1A, B**).

On admission, she was bedridden, unable to resist gravity, and exhibited bladder and rectal disturbances, with a Karnofsky Performance Score (KPS) of 20. Whole-body diffusion-weighted magnetic resonance imaging (WBDWI) revealed neurolymphomatosis relapses of the bilateral trigeminal nerves, cervical/brachial plexus, brachial nerves, lumbosacral plexus, and femoral nerves (**Figure 1D**). Similarly, positron emission tomography-computed tomography (PET-CT) showed ^18^F-fluorodeoxyglucose (FDG) uptake (maximum standardized uptake value [SUVmax] 17.3, **Figure 1C**) in the same region identified on the WBDWI. PET-CT also demonstrated masses with FDG accumulation in the pancreatic head, cecum, abdominal wall, mediastinum, and palatine tonsils (SUVmax 14.0, **Figures 1C, D**). Contrast-enhanced magnetic resonance imaging (MRI) and cerebrospinal fluid examination did not detect central nervous system recurrence.

Intensive treatment regimens were considered intolerable due to her extremely low performance status; therefore, an ibrutinib-based regimen was administered alongside palliative radiation to her left cervical plexus. The 28-day regimen consisted of ibrutinib 560mg/d (day 1–28) + rituximab 375mg/m^2^ (cycle 1: day 1, 8, 15, 22, cycle 2–: day 1) + dexamethasone 40mg (cycle 1: day1, 8, 15, 22, cycle 2–: day 1) + intrathecal methotrexate 15mg/cytarabine 40mg (day 3). After the third course, WBDWI and PET-CT images (**Figures 1E, F**) demonstrated a complete resolution of the neurolymphomatosis lesions, and she regained the ability to walk, achieving a KPS of 60. According to the Revised Response Criteria for Malignant Lymphoma (9), this was consistent with a complete metabolic response (CMR). Adverse events included Grade 2 thrombocytopenia and Grade 3 neutropenia, which were effectively managed with subcutaneous injections of filgrastim and a temporary dose reduction of ibrutinib. However, the disease relapsed, presenting progressive muscle weakness in the masticatory muscles and tingling pains in her teeth and face after the seventh course. PET-CT scans revealed neurolymphomatosis lesions of bilateral trigeminal nerves, brachial nerves, and lumbosacral plexus with SUVmax 19.0. An intra-abdominal soft tissue mass exhibited SUVmax 15.6 (**Figure 1G**). Enhanced MRI also indicated the bilateral lesions of the trigeminal nerves (**Figure 1H**). The progression-free survival with ibrutinib treatment was six months.

The patient’s ability to walk deteriorated again and her KPS dropped to 40. Treatment with epcoritamab was initiated according to the dosage used in the Phase I/II trial EPCORE NHL-1 (10). After the third dose of epcoritamab on day 15, she experienced Grade 1 cytokine release syndrome (CRS) on day 17, which was manageable with tocilizumab. No immune effector cell-associated neurotoxicity syndrome (ICANS) was observed. On day 21, she developed general malaise, and her beta-2 microglobulin (B2MG) level increased from 2.61 to 6.65 mg/L. However, enhanced CT images indicated improvement in the trigeminal nerve lesions. Grade 2 thrombocytopenia and Grade 1 anemia were noted on day 22, but these conditions quickly resolved within a week. No neutropenia was observed. After the fourth dose on day 22, tumor markers and symptoms showed improvement, and tumoral lesions disappeared after the fifth dose, achieving CMR (day 35, **Figures 1I, J**). By day 62, B2MG had improved to 2.28 mg/L. She regained the ability to walk and her KPS improved to 60. Serum IgG levels declined to 255 mg/dL, necessitating multiple subcutaneous IgG supplementations. We continued the treatment for six months; however, the disease unfortunately relapsed in the omentum (SUVmax 12.2, **Figure 1K**). No lymphomatosis lesions were detected, and her performance status was maintained. Given the absence of concerns regarding blood-nerve barrier penetration, she received three courses of salvage chemotherapy with conventional cytotoxic agents (R-ESHAP: rituximab, etoposide, cisplatin, cytarabine, and methylprednisolone). Peripheral blood CD3-positive lymphocyte counts were 721/μL prior to leukapheresis, and T-cell collection was performed between the second and third cycles of R-ESHAP. Three months after the non-lymphomatosis relapse, lisocabtagene maraleucel was administered. Lymphodepletion chemotherapy with fludarabine and cyclophosphamide was given on days -5, -4 and -3. Grade 1 CRS and Grade 1 ICANS were successfully managed with tocilizumab and dexamethasone. Computed tomography demonstrated complete resolution of omental lesions (**Supplementary Figure 2**), indicating that the treatment induced fourth CR. However, she has been experiencing severe cytopenia since approximately day 28 (Grade 4 leukopenia, anemia and thrombocytopenia), for which she is currently receiving granulocyte colony-stimulating factors and blood transfusion.

## Discussion

Most classical cytotoxic chemotherapies are unable to penetrate the blood-brain barrier (BBB) to reach the central nervous system (CNS), although a few limited agents can cross this barrier. In addition to methotrexate and cytarabine, BTKis, which are also small molecules, can pass through the BBB. Ibrutinib, not only being a small molecule (~450 Da), but also has been shown in experimental models to disrupt endothelial tight junction integrity and increase vascular permeability in the BBB (11). Clinical responses to ibrutinib have recently been reported in patients with CNS lymphoma harboring the *MYD88L265P* mutation (12–14). Although not a small molecule, CAR-T cell therapy has also demonstrated efficacy in both primary and secondary CNS lymphoma, probably overcoming the BBB with an underexplored mechanism involving immune activation (15–19). Similarly, clinical responses to another CD20-CD3 bispecific antibody glofitamab have been reported in patients with secondary CNS B-cell lymphoma (20).

In contrast to CNS lymphoma, due to the rarity of neurolymphomatosis, there is limited research on the penetration of anti-lymphoma agents into peripheral nerve lesions. The blood-nerve barrier, like the BBB, is formed by tight junctions between endothelial cells and normally restricts entry of circulating molecules into peripheral nerves (21). However, inflammatory conditions are known to disrupt blood-nerve barrier integrity and permit leukocyte transmigration (22). Neurolymphomatosis is characterized by direct lymphomatous infiltration of peripheral nerves, which likely induces local inflammation and barrier dysfunction. Indeed, a recent article indicated that CAR-T cell therapy was effective in neurolymphomatosis, suggesting that immune effector cells can access peripheral nerve lesions under pathological conditions (7). Because epcoritamab binds CD3-positive T cells, trafficking of activated T cells across inflamed endothelium may facilitate its delivery to peripheral nerve tissue. Although direct evidence is lacking, ibrutinib may similarly penetrate the blood-nerve barrier through mechanisms analogous to those described for the BBB, particularly under inflammatory conditions, given its small molecular size and reported ability to modulate endothelial tight junctions. While direct pharmacokinetic data in neurolymphomatosis are lacking, our observation of CR suggests that BTK inhibitors and CD20-CD3 bispecific antibodies may achieve biologically meaningful concentrations within peripheral nerve lesions.

Patients with neurolymphomatosis often experience impaired physical function due to their neurological symptoms and do not have the necessary physical strength to undergo CAR-T therapy. Previous trials of CAR-T cell therapy for DLBCL have been conducted with patients at Eastern Cooperative Oncology Group Performance Status levels of 1–2 or higher (23–25). In the recent report, CAR-T cell therapy for neurolymphomatosis was administered to patients with a KPS of at least 50 (7). In our case, we had to initiate treatment with a KPS of 20 to 40, prompting us to explore less intensive therapeutic options. The ibrutinib and epcoritamab we utilized were indeed well tolerated. Additionally, once CAR-T therapy is administered, it cannot be removed in the event of a serious adverse event, whereas bispecific antibodies and BTKis have the advantage that they can be discontinued if necessary. However, bispecific antibodies and BTKis have not yet accumulated as much evidence as CAR-T therapy, leading us to consider them as a bridging strategy until the patient regains sufficient strength. Neurolymphomatosis frequently causes rapid neurological deterioration and marked decline in performance status, necessitating urgent disease control. In such settings, CAR-T therapy may not be immediately feasible due to manufacturing time and eligibility constraints. Bridging strategies using bispecific antibodies or BTKis may therefore provide disease stabilization while allowing time for functional recovery and CAR-T preparation.

Ibrutinib has been shown to be effective against MCD-like disease and the ABC phenotype in DLBCL (26, 27). However, in the recent article, three patients with neurolymphomatosis were treated with ibrutinib as a bridging therapy prior to CAR-T cell treatment, with only one patient demonstrating any response (7). While ibrutinib may exhibit activity against *MYD88L265P*-mutant disease with neurolymphomatosis, as observed in our case, its efficacy appears to be limited in duration similar to what has been reported in CNS lymphoma (12–14). Although not confirmed in this case, there is a report indicating that a PIM1 mutation, which is associated with ibrutinib resistance, was detected in a genetic mutation analysis of neurolymphotomatosis (28). This may explain the limited efficacy of ibrutinib despite its MCD-like phenotype. It may be positioned as a bridging therapy rather than a curative approach.

In addition, it is important to note that the six-month duration of CR with epcoritamab was relatively short compared to the EPCORE NHL-1 trial, where the maintenance of CR was 93% at six months (29). This patient carried the *MYD88L265P* mutation and an MCD-like phenotype (non-GCB). Recent findings indicate that the immune microenvironment particularly responsive to bispecific antibodies is characterized by an immune-hot GCB phenotype (30), which may account for the lack of prognosis observed in our case. Recently, there has been a report suggesting that the CD3-CD20 bispecific antibody glofitamab is promising as a bridging therapy for CAR-T (31) and epcoritamab may also be considered as a bridging strategy. Given concerns about T-cell exhaustion caused by bispecific antibodies especially for two weeks after the last bispecific antibody dose (32), determining the appropriate duration of a treatment hiatus prior to T cell collection for CAR-T will be a topic for future consideration.

This report has several important limitations. First, this is a single-case observation, and therefore our findings cannot establish treatment effectiveness but rather demonstrate clinical activity in an individual patient. Second, the clinical response observed during the ibrutinib phase occurred in the context of concomitant therapies, including rituximab, dexamethasone, intrathecal chemotherapy, and localized radiotherapy, making it difficult to isolate the specific contribution of ibrutinib. Third, our discussion regarding drug penetration across the blood–nerve barrier is extrapolated from data on the BBB and remains mechanistic and unproven in neurolymphomatosis. Direct pharmacokinetic measurements within peripheral nerve tissue were not performed. Fourth, comprehensive genomic profiling beyond *MYD88* mutation status was not available, limiting our ability to fully interpret resistance mechanisms or correlate response with molecular features. Finally, the duration of remission following bispecific antibody therapy was relatively short, underscoring that this regimen may not represent a durable standalone strategy.

## Conclusion

In conclusion, this is the first report that epcoritamab is effective in treating neurolymphomatosis. Neurolymphomatosis has not been extensively studied, and further cases need to be documented. BTKis and bispecific antibodies, which demonstrated efficacy in our case, could apply to patients with frailty. These treatments could serve as a bridging strategy toward CAR-T cell therapy, which restore the physical strength deteriorated by neurolymphomatosis.

## Data Availability

The raw data supporting the conclusions of this article will be made available by the authors, without undue reservation.

## Glossary

B2MG: beta-2 microglobulin
BBB: blood-brain barrier
BTKi: Bruton’s tyrosine kinase inhibitor
CAR-T: chimeric antigen receptor T-cell therapy
CNS: central nervous system
CR: complete remission
CRS: cytokine release syndrome
DLBCL: diffuse large B-cell lymphoma
FDG: 18F-fluorodeoxyglucose
GCB: germinal center B-cell
G-CSF: granulocyte colony stimulating factor
HD-MTX: high-dose methotrexate
ICANS: immune effector cell-associated neurotoxicity syndrome
KPS: Karnofsky Performance Score
MRI: magnetic resonance imaging
NL: neurolymphomatosis
OS: overall survival
PET-CT: positron emission tomography-computed tomography
PFS: progression-free survival
Pola-R-CHP: polatuzumab vedotin, rituximab, cyclophosphamide, doxorubicin, and prednisone
R+Dex: rituximab and dexamethasone
R-ESHAP: rituximab, etoposide, cisplatin, cytarabine, and methylprednisolone
SUVmax: maximum standardized uptake value
WBDWI: whole-body diffusion-weighted magnetic resonance imaging
