# Supplementary material for: Case Report: Secondary neurolymphomatosis successfully treated with sequential Bruton’s tyrosine kinase inhibitor and bispecific antibody therapy
Source: Front Oncol. 2026 Mar 12;16:1738551. doi: 10.3389/fonc.2026.1738551 (PMC13017368; doi:10.3389/fonc.2026.1738551)
Supplement: Supplementary file 1 [file DataSheet1.docx]

Supplementary Material

# Supplementary Figures

**
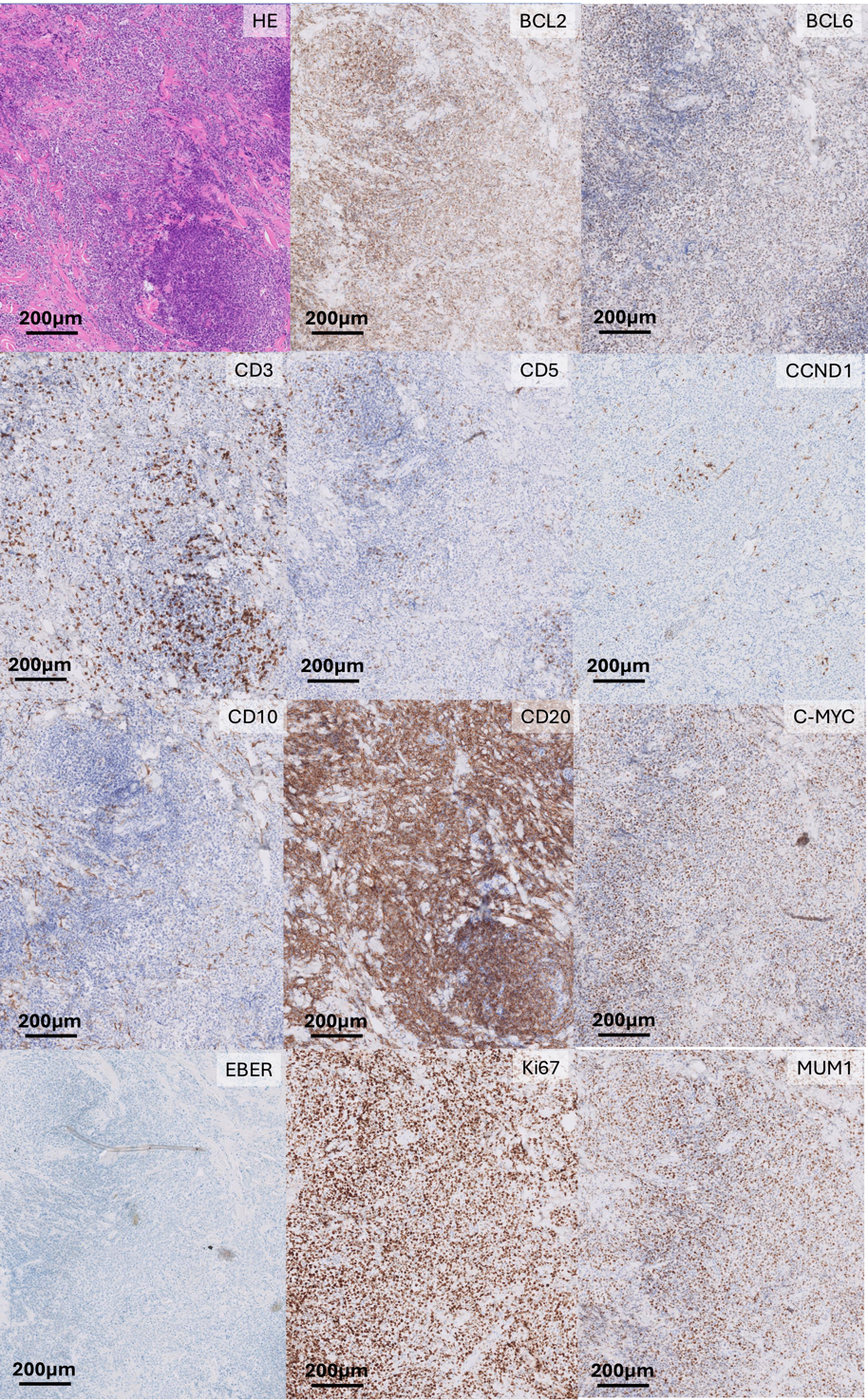
**

**Supplementary Figure 1.** Histology of initial diffuse large B-cell lymphoma

Infiltrated large B-cells in the skin lesion showed CD20+, CD5-, CD10-, BCL2+, BCL6+, MUM1+, C-MYC+, CCND1-, EBER (in situ hybridization)-, Mib-1 index high (80%).


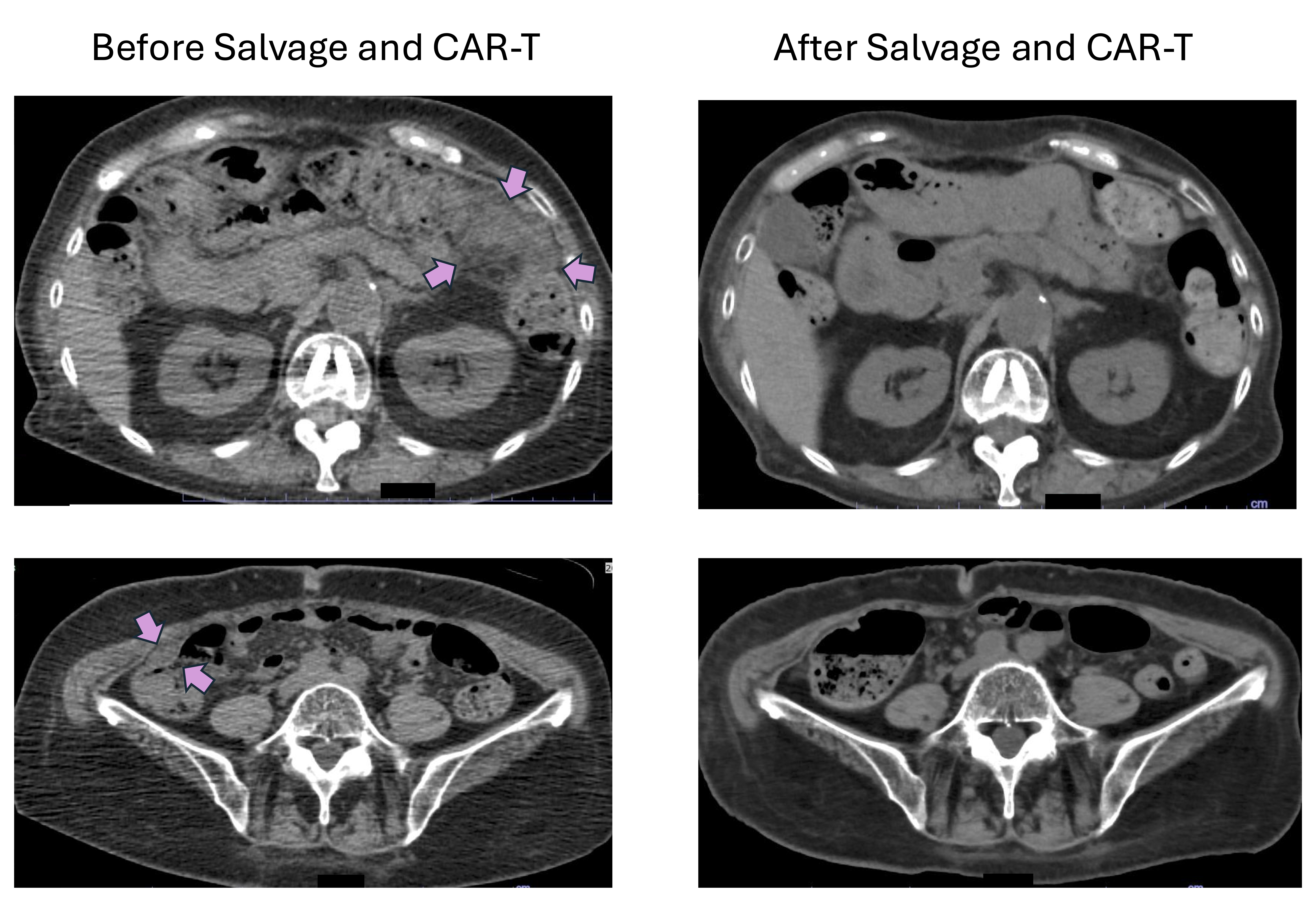


**Supplementary Figure 2.** Resolution of relapsed lesions in omentum.

The non-neulolymphomatosis lesions resolved after salvage chemotherapy (R-ESHAP: rituximab, etoposide, cisplatin, cytarabine, and methylprednisolone) and chimeric antigen receptor T-cell therapy (CAR-T: lisocabtagene maraleucel).
